# The Influence of the Soil Profile on the Formation of the Elemental Image of Grapes and Wine of the Cabernet Sauvignon Variety

**DOI:** 10.3390/molecules29102251

**Published:** 2024-05-10

**Authors:** Zaual Temerdashev, Aleksey Abakumov, Alexan Khalafyan, Mikhail Bolshov, Aleksey Lukyanov, Alexander Vasilyev, Evgeniy Gipich

**Affiliations:** 1 Analytical Chemistry Department, Faculty of Chemistry and High Technologies, Kuban State University, Krasnodar 350040, Russia; temza@kubsu.ru (Z.T.); khaliphyan@kubannet.ru (A.K.); jon.gipich@mail.ru (E.G.); 2 Institute for Spectroscopy Russian Academy of Sciences, Troitsk, Moscow 108840, Russia; mbolshov@mail.ru; 3 North Caucasian Federal Research Center of Horticulture, Viticulture, Wine–Making, Krasnodar 350072, Russia; lykaleks@mail.ru

**Keywords:** geographical origin, ICP-OES, chemometrics, foods analysis

## Abstract

The features for assessing the authenticity of wines by region of origin are studied, based on the relationship between the mineral composition of the wine, the grapes, and the soil profile (0 to 160 cm) from the place of growth of Cabernet Sauvignon grapes. Soil, grape, and wine samples were taken from the territories of six vineyards in the Anapa district of Krasnodar Territory, Russia. Using the methods of ICP-OES, thermal, and X-ray phase analysis, the soils were differentiated into three groups, differing in mineralogical and mineral compositions. The soil samples of the first group contained up to 31% quartz, the second group up to 25% quartz and 19% mixed calcite, and the third group up to 32% calcite and 15% quartz. The formation of the elemental image of the grapes was studied, taking into account the total content and mobile forms of metals in the soil. The territorial proximity of the vineyards did not affect the extraction of elements from the soil into the grape berry, and the migration of metals for each territory was selective. According to the values of the biological absorption coefficient, the degree of transition of metals from the soil to a berry was estimated. For K, Ti, Zn, Rb, Cu, and Fe in all berries, the coefficient was higher than 1.00, which means that the berry extracts contained not only mobile-form, but also difficult-to-dissolve metal compounds. The migration of macro-components from the soil to the berry was low, and amounted to 6–7% for Ca, 0.8–3.0% for Na, and 25–70% for Mg of the concentration of their mobile forms. For all territories, the maximum correlation between metal concentrations in grapes and soil was observed for samples from a depth of 0–40 cm. The discriminant model based on concentrations of Rb, Al, K, Sr, Co, Na, Pb, Ca, and Ni showed the formation of clusters in the territories of vineyard cultivation. The developed model allow the problems of identifying wines by region to be solved with high accuracy, using their elemental image.

## 1. Introduction

Wines with a controlled variety, regional affiliation, and production technology are considered to be original and of high quality [1]. The high popularity of these wines is based on the guaranteed quality, singularity, and uniqueness of the organoleptic characteristics due to the ecological and geographical conditions of the place of origin of the grapes for production, the features of the technology, as well as the manufacturer brand [2]. For their production, grapes grown in a certain zone (area or terroir), indicated on the label, are used according to a strictly regulated technology [3].

Analytical approaches are being developed to control geographical authenticity and gain deeper insights into the chemical composition of wines and their variations. They are based, as a rule, on the study of the mineral and isotopic composition of wines, grapes, and soils corresponding to the area of berry growth [4,5,6,7]. In 2023, the International Organization of Viticulture and Wine (OIV) updated its collection of international methods for the analysis of wine and grape juice, which plays a significant role in the harmonization of analytical techniques and regulates various approaches to analyzing samples [8]. In many wine-producing countries of the world, the mineral composition is used as a tool for identifying the geographical origin of wines [6,7,9,10,11,12,13,14]. This approach makes it possible to determine the quality of wines, identify counterfeits, and also identify wines by name and origin, due to the specificity of the resulting sample profiles [15,16,17,18].

In addition, the mineral content of wine is an important factor in determining the quality of the beverage [19,20,21,22,23,24,25]. K, Ca, Mg, and P are essential for the growth and development of yeast cells during the alcoholic fermentation of grape juice, while Fe, Cu, Si, Zn, and Al play an active role in redox reactions at various stages of winemaking [19]. Mineral components, interacting with amino acids and phenolic compounds, mainly determine the taste properties of wines [20,21]. A content of Al above 5 mg/L in wine leads to the appearance of a metallic taste and the smell of hydrogen sulfide, and a concentration of Zn above 5 mg/L gives an unpleasant odor, roughness, and an astringent–bitter taste [20,22]. Cu and Fe concentrations of more than 1 and 7 mg/L, in addition to a bitter metallic taste, affect the aroma of wine [23]. Elevated concentrations of Al, Fe, and Zn (more than 5 mg/L), and Cu and Ni (more than 1 mg/L) can cause the formation of metallic and colloidal opacities [24]. Excessive metal content in wine can have a negative impact on the health of consumers [25]. OIV has established the maximum permissible concentrations of the following elements in wines: As—0.20 mg/L; Cd—0.01 mg/L; Cu—1.00 mg/L; Pb—0.15 mg/L; Zn—5.00 mg/L; and Ag < 0.1 mg/L [26]. In EU countries, the concentration of Na is also standardized, and should not exceed 60 mg/L, because its high content impairs the harmony of taste—the wine acquires “soapy tones” [27].

The main supply of elements to grapes and wine occurs from the soils of the vineyard through the roots of the grapevine [28,29]. Soil is an important nutrient medium in which the development of underground organs of the grapevine (root trunk, root system) takes place [30]. The grapevine, participating in geochemical processes, selectively absorbs the elements it needs from the soil in quantities corresponding to its physiological and biochemical needs [21,22,23,24,25,26,27,28,29,30,31,32,33]. Various macroelements enter the plant from the soil, such as K (contained in the tissues of grapes), Ca (stimulates the growth and development of the plant as a whole), Na, Mg, and microelements—Fe, Cu, Zn, Co, Mn, etc.—affecting various enzymatic and other biological processes of the grapevine [34]. Nutrients are most actively absorbed from the soil when the berries begin to ripen. A higher content of elements is typical for the solid parts of the berry—the skin, cell walls, pulp, and seeds [35,36].

The authors of [37] assessed the nature of the supply of metals in the soil–grape chain, taking into account three extraction forms of elements from soils: total, acid-soluble, and mobile. In addition to mobile forms, the grapevine can also extract poorly soluble compounds from the soil [38], and each grape variety forms its own individual elemental image due to the different natures of metal absorption.

The chemical composition of plants is largely formed by the component composition of soils but does not replicate it. The issue of “contamination” of vineyard soils is also relevant [39] because soils are often severely degraded and require the application of various root and foliar fertilizers that improve the growth and development of the plant and its fruits [40,41]. The application of organic and mineral fertilizers to vineyards has different effects on the chemical composition and quality of berries, and the wine produced from them [42,43]. An excess of nitrogenous substances increases the acidity of grapes; as a result, wines are prone to cloudiness and microbial diseases, and their sensory properties are characterized by a less pure taste and aroma [44,45]. Potassium fertilizers reduce the acidity of grapes, increasing the extraction of wines and improving the quality of red wines [45]. Excessive application of phosphorus fertilizers also affects the quality of grapes, reducing the total amount of soluble solids, anthocyanins, and polyphenols, as well as increasing the total titratable acidity in the berries and must [46,47]. Thus, the ratios of mineral nutrients in the finished wine have a complex, indirect, and distant correlation with the geological minerals in the vineyard. Therefore, to detect falsification or establish the regional origin of wine, many researchers select elements of the mineral composition that are least dependent on external influences for a specific geographical area [48,49].

The number of publications on the influence of soil features on the quality of wine has been growing in the last several years [50,51,52]. However, they do not provide a detailed explanation of the mechanisms behind these processes. The most likely means by which soil can influence the composition of wine is through the mineral nutrition of the vine. This is achieved by the availability of nutrients in the soil that are then absorbed by the vine, thus providing it with the necessary minerals for its growth and development [29]. In arid climates, the availability of nutrients in the topsoil decreases during the growing season, becoming a limiting factor for their delivery to plant roots. Soil structure also plays a significant role in determining how many nutrients are available for uptake by plant roots. Nevertheless, a traceable sequential correlation between the elemental composition from soil to wine has not been identified and the influence of soil chemical features on the formation of the elemental composition of various grape varieties has not been established.

The purpose of this work is a spectrometric and chemometric study of the influence of the soil profile on the formation of the elemental image of grapes and wine of the Cabernet Sauvignon variety grown on the territory of six vineyards of different elemental composition in the Anapa region of the Krasnodar Territory, Russia.

## 2. Results and Discussion

Samples of Cabernet Sauvignon grapes were grown on different types of soils and were selected from six vineyards with similar rootstocks located on the Black Sea coast in the Anapa region of the Krasnodar Territory, Russia. The close location of the vineyards to the Black Sea allows one to avoid sharp negative temperatures in winter, and makes the climate calmer and “mild” the rest of the time. Taking into account these unique conditions for growing grapes, we studied the distribution and relationship between the total metal content and mobile metal forms in soil profiles up to a depth of 160 cm. We used ICP-OES, X-ray fluorescence, and X-ray diffraction to analyze the composition of the soil profile in the vineyards.

### 2.1. Component Composition of Soil Profiles of Vineyards

**Figure 1:**
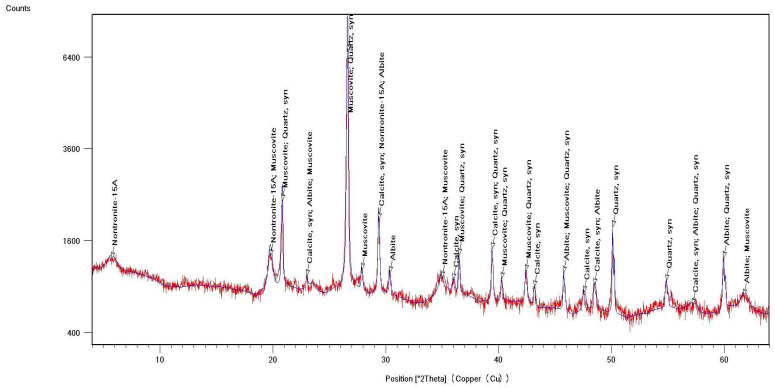
X-ray diffraction pattern of a soil sample from Vinogradny (first group).

**Figure 2:**
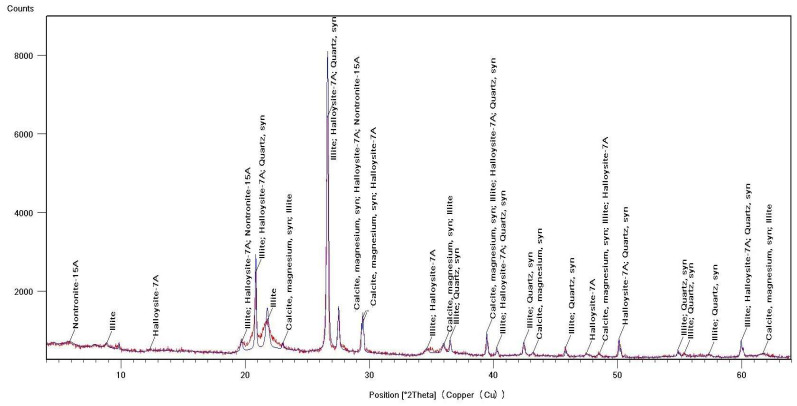
X-ray diffraction pattern of a soil sample from Anapa (second group).

**Figure 3:**
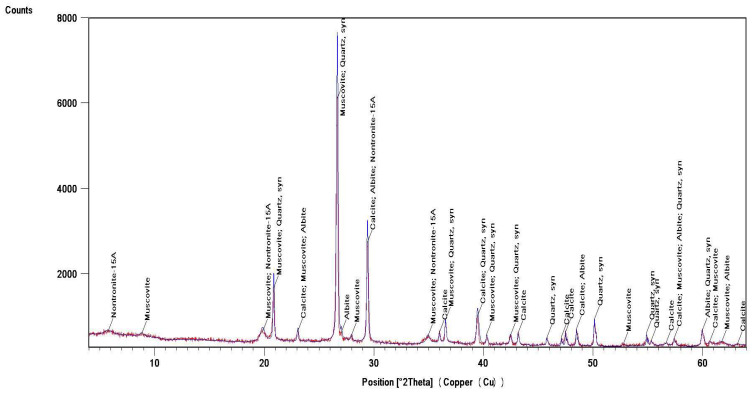
X-ray diffraction pattern of a soil sample from the village Haykadzor (third group).

Identification of the component composition of soils was carried out using PDW software version 4.0 and the Crystallographica Search-Match software (Oxford Cryosystems Ltd., Long Hanborough, UK), which are integrated into the hardware and software complex of the diffractometer. X-ray phase analysis allowed us to differentiate the soil samples into three groups according to their phase profiles: the first group included soils from the Vinogradny and Yurovka vineyards (Figure 1), the second from Anapa and Raevskaya (Figure 2), and the third from Haykadzor and Gostagayevskaya (Figure 3). Qualitative powder diffraction analysis revealed the presence of crystalline phases identified from the PDF-2 database: quartz (Ref. Code 00-046-1045), dolomite (Ref. Code 01-089-1304), calcite (Ref. Code 01-081-2027), feldspar (albite) (Ref. Code 00-003-0451), nontronite (Ref. Code 00-029-1497), muscovite (illite) (Ref. Code 01-070-3754), and vermiculite (Ref. Code 01-074-1732). The clay fraction in the soil samples was predominantly represented by mica (Ref. Code 00-003-0849). The soil samples of the first group (Vinogradny) were distinguished by a lower content of calcite, or its complete absence; it contained more silicate phases—quartz (Ref. Code 00-046-1045), albite (Ref. Code 01-070-3752), and vermiculite (Ref. Code 01-074-1732). The semi-quantitative phase analysis in soil samples of the first group made it possible to identify quartz (Ref. Code 00-046-1045) up to 31%. In the soil samples of the second group, up to 25% quartz (Ref. Code 00-046-1045) and 19% mixed calcite (Ref. Code 01-089-1304), as well as hydromica (Ref. Code 01-070-3754) and smectites (Ref. Code 00-029-1497), were identified. In the soil samples of the third group, calcite (Ref. Code 01-081-2027) was contained in up to 32%, quartz (Ref. Code 00-046-1045) in up to 15%, and feldspars (Ref. Code 00-003-0451) and hydromica (Ref. Code 00-003-0849) were also identified.

To clarify the component composition, thermal analysis of the soil samples was conducted in the temperature range of 20–1000 °C. When interpreting the thermal analysis data, we relied on data from a well-established reference book on thermogravimetric systems of minerals and their use in geological applications [53], as well as on studies of the connection between geology, soil evaluation, and vineyard terroir [54].

**Figure 4:**
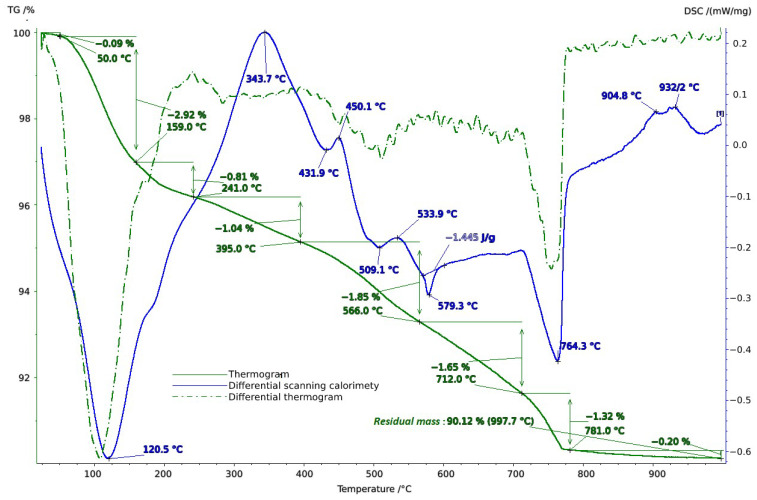
Thermogram of soil sample from Vinogradny.

**Figure 5:**
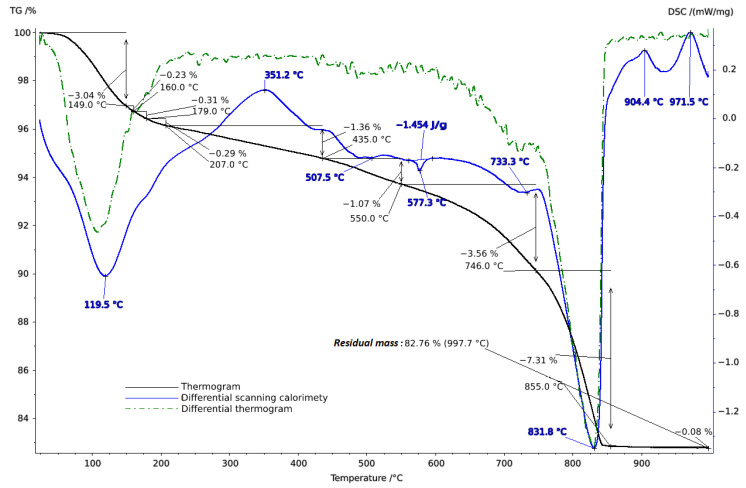
Thermogram of soil sample from Anapa.

**Figure 6:**
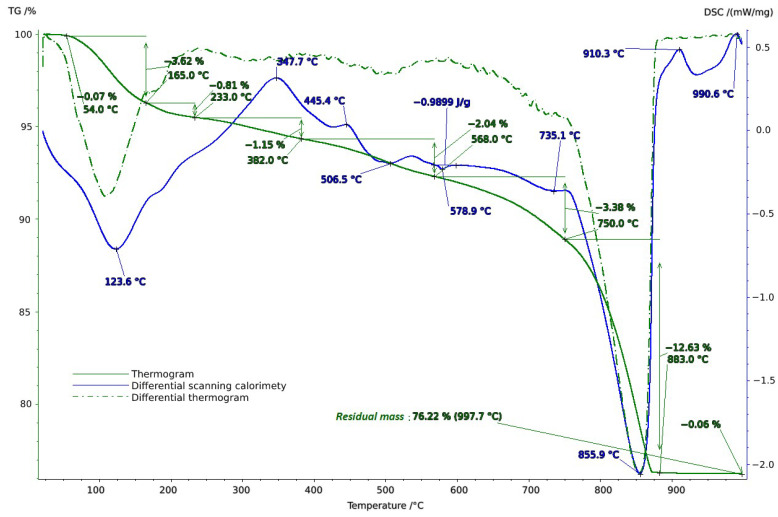
Thermogram of soil sample from Haykadzor.

Thermal studies made it possible to clarify the main stages in the studied soil samples based on their temperature transformations. The thermal analysis curves confirm the X-ray phase analysis data on the composition of the soil samples, which contain mainly clay minerals and silicates (Figure 4, Figure 5 and Figure 6). The loss of weight in all thermograms up to 240 °C is apparently due to endothermic dehydration, caused by the loss of absorbed moisture and interlayer water in smectites (illite, ntrontite) [53]. The endothermic effect at 579 °C on the DSC curve is associated with a phase transition in quartz, which is more pronounced in the soil sample from Vinogradny village (Figure 4). The dehydroxylation of vermiculites in the soil samples takes place in a range from 450–750 °C, and the rate of this process is very slow, making it difficult to observe as a distinct peak [53]. Wide variations in vermiculate composition result in significant differences in dehyroxylation processes. On the TG curve for samples from Anapa and Haykadzor, we observed mass losses of 7.3% and 12.6%, respectively. These losses are characteristic of carbonate-containing sediments (calcite and/or dolomite). The losses occur in the temperature range of 750–880 °C, which corresponds to the decomposition of calcite with the release of CO_2_. This indicates that the thermal decomposition process has destroyed the calcite structure, which was formed during weathering. The usual one-step reaction has turned into a two-step process [53]. The results of the thermal and X-ray phase studies confirm the possibility of a transition between silicate and carbonate soils in neighboring vineyards. One author states that the content of the main oxides in soil can be directly related to the presence of carbonate and silicate clay minerals [54].

**Figure 7:**
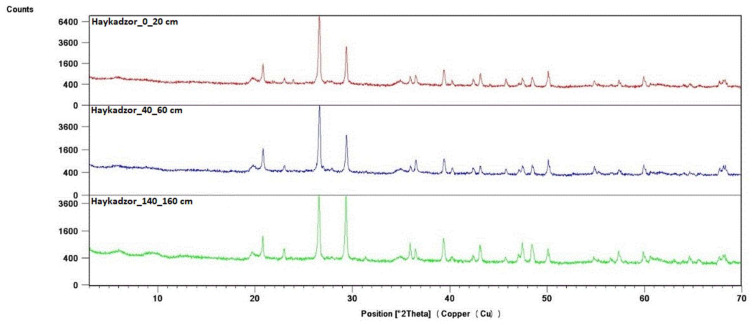
X-ray diffraction patterns of the soil profile of the vineyard in Haykadzor.

The root system of the grapevine is capable of covering different layers of soil and subsoil, reaching up to 10 m in depth. In this case, the greatest interest is in the soil horizons of the active absorption zone, where the bulk of the roots is located. According to various estimates, these layers are located at a depth from 20 to 80 cm [55,56,57,58]. For example, the results of the X-ray phase analysis of the soil profile of a vineyard from the territory of Haykadzor showed that the active absorption zone for this territory was located at a depth of 40–60 cm (Figure 7). The X-ray diffraction patterns from other samples showed a similar composition across the sampling horizons for each sampling location, so further studies were carried out for a depth of 40–60 cm.

Data on the elemental composition (total content and mobile forms) of soils, grapes, and wine (average value ± combined uncertainty) are summarized in Table 1, Table 2 and Table 3 considering the classification of soils into groups.

For soils classified in the first group, the total contents of most elements are quite close—Ca, K, Ba, Mn, Cd, Co, Cr, Li, Rb, V, Ni, Pb, and Zn (Table 1). The concentrations of mobile forms of elements are significantly closer, which differ only for the contents of Na and Co. A similar correlation in elemental composition was observed for the soils assigned to groups 2 and 3 (Table 2 and Table 3). The validity of dividing soils into groups according to their mineralogical composition and considering the main phases can be asserted.

**Figure 8:**
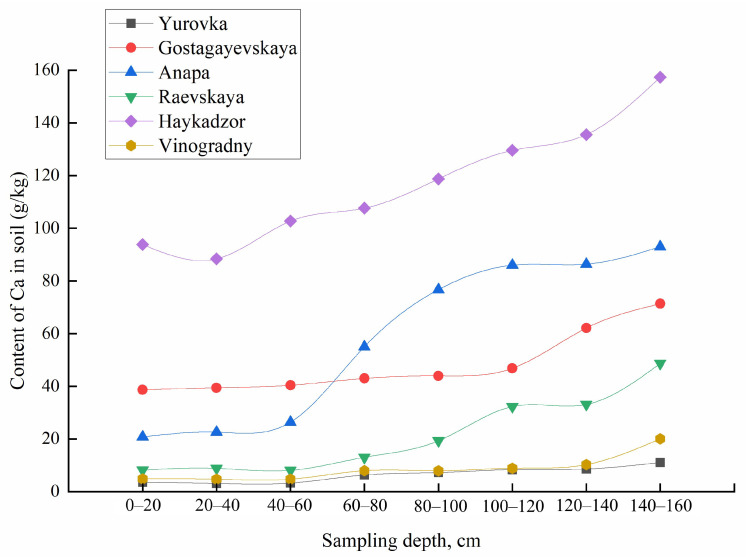
Total content of Ca in the soil at different sampling depths.

**Figure 9:**
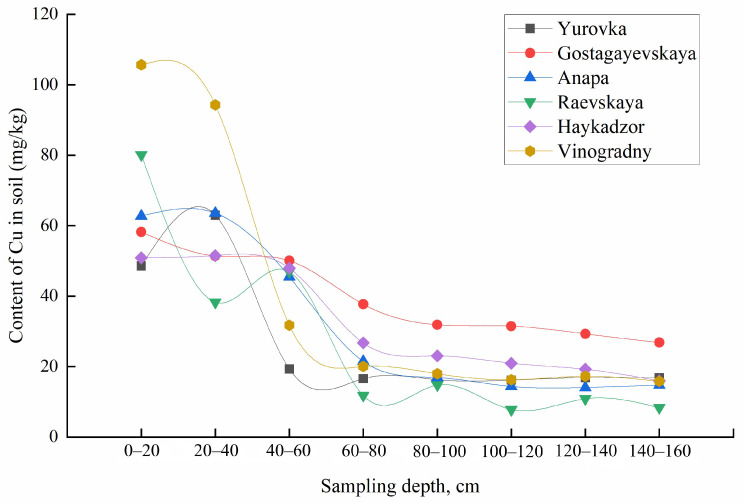
Total content of Cu in the soil at different sampling depths.

The obtained functions of the distribution of metals over the sampling depth differ for the mobile forms and gross contents of most elements. With increasing sampling depth within the same occurrence form, metals also behave differently. The concentrations of mobile forms of K and Cu decrease with increasing sampling depth for all regions, meanwhile for Ca, Mg, Na, Fe, and Sr, an inverse correlation was observed. The total content of Ca, Na, Mg, Rb, Sr, and Ti increased; for K, Cu, Pb, Fe, Zn, and Mn, it decreased; and the content of Cd, Co, Cr, Li, and Ni remained constant with increasing soil sampling depth (Figure 8 and Figure 9). The concentrations of Al, Ba, and V varied in different ways with an increase in the depth of soil sampling, depending on the area of cultivation.

### 2.2. The Correlation between the Elemental Composition of Soil, Grapes, and Wine

It should be noted that the grape variety under study is cultivated in one administrative district. The soil samples studied belong to calcareous types, but the average values of the total content and mobile forms of metals in the soil groups were found to be different. The elemental composition of grape berries, formed by the root system and taking into account the rootstock, was more homogeneous compared to the metal content in the soils (Table 1, Table 2 and Table 3). A direct correlation between the transition of an element to a grape berry and the gross content or mobile form of that metal in soils was not observed. This fact can be explained by the intraspecific differences in metal concentrations in soil and berries, which are due to the grape variety’s natural ability to select elements from the soil and transfer them to the aboveground parts [59]. The transfer of metals from soil to grapes was assessed by the values of the biological absorption coefficient—the ratio of the concentrations of metals in the berry to the mobile forms of metals in the soil at a depth of 40–60 cm (Table 4).

The data obtained show that, despite their territorial proximity, the migration of elements from the soil into grapes is unique for each cultivation area. K, Ti, Zn, Rb, Cu, and Fe have the highest migration coefficients for all territories, then, in descending order, Mg, V, Sr, Al, Na, Co, Cr, Ba, Mn, Li, Ca, Ni, Pb, and Cd.

Only about 6–7% of mobile Ca is taken up by grape berries. For Na, this figure is 0.8–3.0%. For Mg, it is 25–70%, and so on. This seems to be due to the natural ability of grape varieties to select different elements from the soil. Cr, meanwhile, is practically not taken up by grape berries, and the element does not move well from the soil to the aboveground plant parts [60].

Despite the macro contents of Ca, Na, and Mg in soils, their migration into berries turned out to be significantly lower in comparison to other elements. A disproportionate increase in the contents of K and Zn in the grape samples should be noted, regardless of the territory of grape cultivation and the concentration of their mobile forms in the soil, especially in terms of K. The disproportionate transfer of MF elements from the soil to the berry confirms the fact that the grapevine extracts from the soil are not only mobile forms, but are also more sparingly soluble compounds.

For all six territories in the soil–berry chain, a decrease in the correlation between the concentrations of mobile-form metals in soil and grapes was observed with an increase in sampling depth (Table 5). The gross content is characterized by a discontinuous decrease in correlations in Yurovka and Anapa, while in other territories, the correlations remain relatively stable with increasing sampling depth.

The greatest correlation between the concentrations of metals in grapes and soil for all territories appeared at a depth of 0–40 cm. At the same time, the correlation between the elemental composition of soil and grapes turned out to be weak and statistically insignificant. The lack of a strict correlation can be explained by the fact that the metals absorbed by the plant from the soil are distributed differently in parts of the grapevine.

Strong correlations were observed in the berry–wine chain; the correlation coefficients for all territories are close to 1 (R^2^ > 0.996), and a positive sign means that the higher the metal concentration in the berry, the higher its content in the wine. This statement is true, provided that technological processes do not interfere with the processing of wine materials in such a way that they significantly alter its “elemental composition” [61,62,63].

### 2.3. Establishing the Regional Origin of Grapes

To establish regional differences/similarities in the grape samples in terms of total metal contents, discriminant analysis was used. Predictors (independent variables) of the discrimination model are the concentrations of metals in grapes (15 samples for each region). The grouping (dependent) variable is the name of the grape-growing area. Discriminant analysis allows predictive models to be built for establishing the belonging of objects to given classes, but it can also be used to identify and demonstrate trends, similarities, and differences between classes of objects by reducing the dimension of space by calculating canonical scores and then building a scatterplot of canonical scores in the Root1 and Root2 coordinate system.

To implement the discrimination process, we chose a step-by-step method with an exception, which involves the automatic exclusion of predictors that are redundant for discrimination. Due to the low content of Cd in the studied objects, this metal was not included in the discrimination model. Then, 10 elements (Mg, Ba, Mn, Ti, Cr, Li, Cu, V, Zn, and Fe) were eliminated sequentially in 10 steps, and the 9 remaining elements are presented in the final table: Table 6. A small value of Wilks’ lambda (0.0000) in the upper information part of the table indicates successful discrimination. This means that Cabernet Sauvignon grapes within one cultivation area form regional clusters and are similar, while grapes from different areas are heterogeneous in terms of metal content.

The columns “Wilks’ Lambda” and “Partial Lambda” in Table 6 show the role of elements in the discrimination model. For example, the higher is the Wilks’ Lambda value and the lower Partial Lambda value, the higher is the contribution of the variable in the discrimination model. The elements in the table are arranged in descending order by Wilks’ lambda; therefore, Rb has the largest contribution to discrimination, while Ni has the lowest. At the same time, all elements in the discrimination model are statistically significant—the significance levels of the *p* (*p*-value) of the Fisher criterion (F-remove) are less than 0.05.

**Figure 10:**
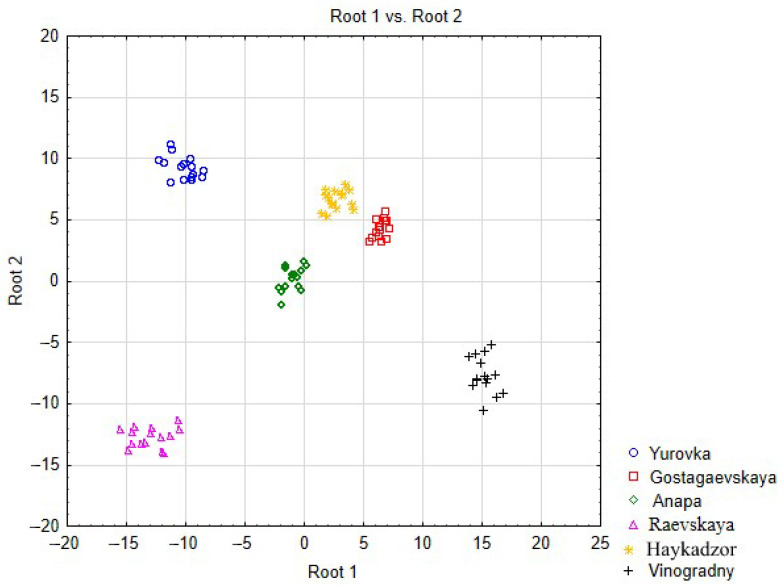
Scatter diagram of the canonical values of grapes.

A graphic illustration of the presence of a regional cluster structure is the scatter diagram of canonical values (Figure 10), in which grape samples are depicted as points on a plane of different configurations and colors depending on their belonging to the region of a vineyard. The diagram allows objects in a multidimensional space to be transferred into a space of lower dimensions—onto a plane—while maintaining the order of distances between objects. Using the diagram, one can judge the homogeneity of groups and the degree of similarity/difference between them through distances according to the principle that the smaller the distance, the greater the similarity. Figure 10 shows that the grape samples from each cultivation area are mainly localized in their specific parts of the plane, forming groups of similar objects—clusters.

## 3. Materials and Methods

### 3.1. Objects of Research

In this work, Cabernet Sauvignon grape samples grown on various types of soil and selected from six vineyards with similar rootstocks in the Anapa district of the Krasnodar Territory, Russia, were studied. Grapes were harvested for processing into wine materials upon reaching technical maturity—a mass concentration of sugars of at least 170 g/L, titratable acids of 6–9 g/L, and an amount of coloring substances of 2.5–2.8% by weight of the raw skin. The processing of grapes and the production of wine materials were carried out in accordance with the general rules and technological instructions for wine production [64]. Wine materials were prepared using the fermentation of grape pulp with active dry yeast Lalvin EC 1118 (Lallemand, Montreal, QC, Canada). The fermentation process was carried out for 7 days, with the temperature kept between 28 and 32 degrees Celsius. During this time, “Ultrasulf S” (Perdomini-IOC, San Martino Buon Albergo, Italy) was added at a rate of 160 g per ton (corresponding to 100 mg/L of SO_2_) to ensure proper sulfation. After fermentation, the grape pulp was pressed using a pneumatic press (Busher Vaslin, Chalonnes-sur-Loire, France), followed by the separation of wine. After the fermentation of sugars and removal of yeast sediment, the wine materials were treated again with “Ultrasulf S”, this time at a dosage of 50 mg/L SO_2_. The grapes used for this wine were harvested in 2022, and the resulting wine can be described as a young wine. The wine was not processed with bentonite prior to the research, nor was it filtered. The clarification was achieved through natural means. The main physico-chemical characteristics of the wine samples studied are presented in Table 7.

**Figure 11:**
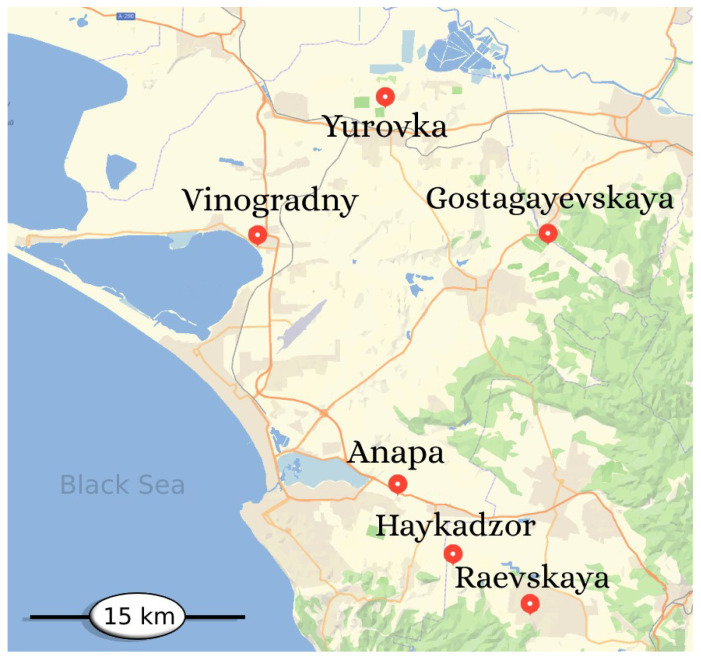
Geographical location of vineyards.

Cabernet Sauvignon grapevines were cultivated on different types of soils, so the distribution of the total content and mobile forms of metals and their correlations at depths of 0–160 cm in soil profiles were studied. Soil samples were taken during the ripening period of berries using the envelope method from depths of 0–20, 20–40, 40–60, 60–80, 80–100, 100–120, 120–140, and 140–160 cm. The geographical location of the vineyards is shown on the map (Figure 11).

### 3.2. Materials and Reagents

For experimental studies, 25% aqueous ammonia, 33% hydrogen peroxide, 99.7% glacial acetic, and 69% nitric acids were used (all of high-purity production, PanReac AppliChem, Darmstadt, Germany). To establish the elemental composition of the samples, multielement calibration solutions were used, which were prepared using single-element standard solutions of metals with a concentration of each element of 1000 mg/L—Al, Ba, Ca, Cd, Co, Cr, Cu, Fe, K, Li, Mg, Mn, Na, Ni, Rb, Si, Sr, Ti, and Zn (Inorganic Ventures, Christiansbur, VA, USA).

### 3.3. Multielement Analysis

Analytes in soils, grapes, and wines were quantified by inductively coupled plasma–optical emission spectrometry using an iCAP-7400 DUO spectrometer (Thermo Scientific, Waltham, MA, USA) [37]. The analysis conditions are summarized in Table 8. The analyzed solutions were prepared using deionized water with a maximum resistivity of 18.2 MΩ cm^−1^ obtained in a sub-distillation installation DuoPUR (Milestone, Milan, Italy).

X-ray diffraction analysis of the BT samples was conducted on a Shimadzu XRD-7000 X-ray diffractometer (Shimadzu, Kyoto, Japan) [54]. Analysis conditions were as follows: Cu K_α_ radiation (1.54 Å), Ni filter, operating voltage of 40 kV, 30 mA, angle range of 3–70 degrees, and scan speed of 1 degree/min.

Thermal analysis of soils was carried out on an STA-409 PC Luxx derivatograph (Netzsch, Selb, Bavaria, Germany) [54]. Thermal analysis conditions were as follows: temperature range of 30–1000 °C, recording in an air atmosphere in platinum crucibles at a heating rate of 10 °C/min.

### 3.4. Determination of Various Forms of Elements in Soils

On the surface of the root hairs of the grapevine, special conditions are created for the active influence of the plant on sparingly soluble soil compounds for their extraction [29]. This fact was taken into account when studying the nature of the supply of metals in the soil–grape chain. The influence of the supply of metals in the soil–grape chain was assessed by the degree of extraction of the gross content of elements from soils, as well as by the extraction of elements by an ammonium acetate extract, which has close acidity to the soil solution formed on the surface of plant roots [65].

### 3.5. Pretreatment of Soil Samples for Analysis

To determine the total content (TC) of elements, soil samples were digested using an Ethos 1 microwave digestion system (Milestone, Milan, Italy) using the method described in [31]. For this, 0.50 g of soil and an oxidizing mixture containing 5.0 mL of concentrated HF, 3.0 mL of concentrated HNO_3_, and 1.0 mL of concentrated HCl were placed in an autoclave. To digest the samples, the analyzed mixture was gradually heated to 200 °C for 25 min, and then kept at 200 °C for 5 min in the reaction chamber. To avoid losses of volatile elements, the autoclaves were opened at temperatures below 40 °C; then, the contents of the autoclave were transferred to a 50 mL polypropylene volumetric flask and brought up to the mark with deionized water. ICP-OES analysis of soil samples was carried out using an HF-resistant sample injection system, including a PFA-ST nebulizer (Thermo Scientific, Waltham, MA, USA), a cyclone-type PFA spray chamber (Glass Expansion, Melbourne, Australia), and a 2.0 mm corundum injector (Glass Expansion, Melbourne, Australia).

The extraction of mobile forms (MF) of elements from soils was carried out using an ammonium acetate extract, which is close in acidity to the soil solution [38]. A 1.00 g sample of soil was placed in a small conical flask, 20 mL of ammonium acetate buffer solution (pH 4.8) was added, and the solution was stirred for 1 h on an orbital laboratory shaker. The next day, the sample was filtered into a 25 mL volumetric flask through a “blue” tape filter and brought to the mark with an ammonium acetate buffer solution.

The completeness of the opening of soil samples and the correctness of the results of element quantification were checked using CRM SADPP-10 (mobile forms) and CRM SDPS-2 (total content) (Table 9).

The elements in grapes were quantified according to the method in [37]. The digestion of grapes before analysis was carried out using an Ethos 1 microwave system (Milestone, Italy). The berries were washed with distilled water and dried; then, 2 g of grape was placed in an autoclave. Digestion of the sample was carried out at elevated pressure using an oxidizing mixture consisting of 5.0 mL of concentrated HNO_3_, 1.0 mL of H_2_O_2_, and 4.0 mL of deionized water. The sample digestion program included gradual heating to 200 °C for 10 min (stage 1) and maintaining the reaction chamber at 200 °C for 10 min (stage 2). Autoclaves were opened at temperatures below 40 °C to avoid losses of volatile elements. Then, the content of the autoclave was transferred to a 25 mL flask and brought to the mark with deionized water. After sample pretreatment, elemental analysis was carried out using ICP-OES.

The preparation of wines to establish elemental composition consisted of a 15-fold dilution of samples with 2% nitric acid considering the data [6,66]. After that, ICP-OES analysis was performed.

### 3.6. Statistical Analysis

The distribution and identification of correlations between the elemental composition of soils, grapes, and wine were studied using STATISTICA (v.13) software (StatSoft, Hamburg, Germany) [67]. Correlation between metal concentrations was assessed using the value of the Pearson correlation coefficient *r*. When |*r*| ≤ 0.25, the correlation was considered weak; 0.25 < |*r*| ≤ 0.75—moderate; and |*r*| > 0.75—strong. The cluster structure of the mobile and bulk metals relative to their concentrations in soil, grapes, and wine was studied by discriminant analysis.

## 4. Conclusions

The influence of the soil profile on the elemental image of grapes and Cabernet Sauvignon wines was studied. Soil and grape samples were taken from the territory of six vineyards of the Anapa district of Krasnodar Territory, Russia. The studied soil samples, according to X-ray phase analysis, belonged to calcareous types. The data from the elemental, thermal, and X-ray phase analyses of soil samples allowed the soils to be differentiated into three groups, differing in mineralogical and elemental compositions. The samples of the first group were characterized by a high quartz content (up to 31%), the second group contained up to 25% quartz and 19% mixed calcite, and the third group contained up to 32% calcite and 15% quartz.

To establish correlations between the elemental composition of the soil from different depths (0–160 cm) and grape berries, data obtained by the ICP-OES method were used. In the soil–berry chain, there was a decrease in the correlation between the concentrations of mobile forms of metals in the soil and in grapes with an increase in the depth of sampling for all six territories. The greatest correlation between metal concentrations in grapes and soil was manifested at a depth of 0–40 cm for all territories. At the same time, the coefficients of correlation of elemental compositions between grape berries and wine were close to one (R^2^ > 0.996) for all samples.

The territorial proximity of vineyard cultivation did not affect the nature of the extraction of elements from the soil into the grape berry. The migration of elements from the soil to the grape berry was selective for each territory. Despite the high content of mobile forms of Ca, Na, and Mg in soils, their migration to the berry turned out to be at the levels of 6–7%, 0.8–3.0%, and 25–70%, respectively. The coefficient of biological absorption of K, Ti, Zn, Rb, Cu, and Fe in berries from all vineyards turned out to be higher than 1.00. This character of the transition of mobile forms of elements from the soil to the berry confirms that the vine extracts contained not only mobile forms from the soil, but also more insoluble compounds. 

Discriminant analysis of the metal content in grapes showed the formation of clusters depending on the cultivation area. This allowed us to solve the problems of identifying wines by their regional affiliation. The discrimination model of the regional cluster structure of the grapes was built according to the following predictors: Rb, Al, K, Sr, Co, Na, Pb, Ca, and Ni (in decreasing order of contribution to the model).

## Figures and Tables

**Table 1:** Data on the elemental composition of soils, grapes, and wine, classified by their qualitative and quantitative phase composition as group 1.

Element	Yurovka				Element	Vinogradny			
	MF ^1^, mg/kg	TC ^2^, mg/kg	Grape, mg/kg	Wine, mg/L		MF ^1^, mg/kg	TC ^2^, mg/kg	Grape, mg/kg	Wine, mg/L
**Ca**	2800 ± 800	3330 ± 910	200 ± 40	59 ± 9	**Ca**	3300 ± 900	4700 ± 1420	220 ± 40	64 ± 10
**Na**	1600 ± 530	27,100 ± 8220	4.8 ± 1.0	10 ± 2	**Na**	54 ± 11	9200 ± 2740	16 ± 3	24 ± 3.6
**Mg**	4400 ± 1320	6930 ± 2120	100 ± 20	68 ± 10	**Mg**	402 ± 121	2400 ± 710	110 ± 20	62 ± 9.3
**K**	112 ± 32	6110 ± 1820	1860 ± 370	702 ± 105	**K**	123 ± 41	6400 ± 1920	3230 ± 650	1270 ± 191
**Al**	71 ± 22	38,100 ± 11,200	4.0 ± 0.8	0.440 ± 0.066	**Al**	62 ± 21	19,100 ± 6340	2.5 ± 0.5	0.440 ± 0.066
**Fe**	8.0 ± 2.3	30,400 ± 9300	5.3 ± 1.0	5.150 ± 0.770	**Fe**	5.0 ± 1.2	20,300 ± 6030	5.2 ± 1.04	4.740 ± 0.711
**Ba**	38 ± 11	149 ± 45	1.4 ± 0.3	0.066 ± 0.010	**Ba**	46 ± 14	168 ± 50	1.5 ± 0.3	0.088 ± 0.0132
**Mn**	19 ± 6	593 ± 178	0.92 ± 0.18	0.704 ± 0.106	**Mn**	29 ± 9	430 ± 129	1.5 ± 0.3	1.020 ± 0.153
**Sr**	15 ± 4	93 ± 28	2.0 ± 0.3	0.570 ± 0.086	**Sr**	12 ± 4	42 ± 12	2.0 ± 0.4	0.360 ± 0.054
**Cd**	0.020 ± 0.006	0.44 ± 0.13	<MLOQ	<MLOQ	**Cd**	0.02 ± 0.01	0.32 ± 0.09	<MLOQ	<MLOQ
**Co**	0.08 ± 0.024	12 ± 4	<MLOQ ^3^	<MLOQ	**Co**	0.02 ± 0.01	13 ± 4	<MLOQ	<MLOQ
**Cr**	0.13 ± 0.04	73 ± 22	<MLOQ	<MLOQ	**Cr**	0.11 ± 0.03	82 ± 24	0.016 ± 0.003	<MLOQ
**Li**	0.31 ± 0.09	27 ± 8	0.012 ± 0.002	0.011 ± 0.002	**Li**	0.31 ± 0.09	28 ± 8.4	0.016 ± 0.003	0.014 ± 0.002
**Rb**	1.5 ± 0.4	62 ± 18	5.6 ± 1.1	5.280 ± 0.790	**Rb**	1.6 ± 0.5	71 ± 21	1.3 ± 0.3	1.710 ± 0.260
**Ti**	0.38 ± 0.11	353 ± 106	0.9 ± 0.2	0.043 ± 0.007	**Ti**	0.33 ± 0.09	231 ± 69	1.1 ± 0.2	0.055 ± 0.008
**V**	0.04 ± 0.01	67 ± 20	0.028 ± 0.006	<MLOQ	**V**	0.05 ± 0.02	75 ± 22	0.021 ± 0.004	<MLOQ
**Ni**	1.2 ± 0.4	35 ± 10	0.026 ± 0.005	0.114 ± 0.017	**Ni**	1.1 ± 0.3	35 ± 10	0.022 ± 0.004	0.140 ± 0.021
**Cu**	0.33 ± 0.11	19 ± 6	0.91 ± 0.18	0.131 ± 0.020	**Cu**	0.36 ± 0.11	32 ± 10	0.90 ± 0.18	0.068 ± 0.010
**Pb**	0.39 ± 0.12	8.9 ± 2.7	0.03 ± 0.01	0.0043 ± 0.0007	**Pb**	0.50 ± 0.15	8.1 ± 2.4	0.03 ± 0.001	0.068 ± 0.010
**Zn**	0.29 ± 0.09	54 ± 16	1.1 ± 0.2	0.140 ± 0.021	**Zn**	0.16 ± 0.05	55 ± 16	0.82 ± 0.16	0.217 ± 0.033

^1^ MF—mobile-form element. ^2^ TC—total content element. ^3^ *MLOQ—method limit of quantification.*

**Table 2:** Data on the elemental composition of soils, grapes, and wine, classified by their qualitative and quantitative phase composition as group 2.

Element	Raevskaya				Element	Anapa			
	MF ^1^, mg/kg	TC ^2^, mg/kg	Grape, mg/kg	Wine, mg/L		MF ^1^, mg/kg	TC ^2^, mg/kg	Grape, mg/kg	Wine, mg/L
**Ca**	5310 ± 1630	8120 ± 2440	310 ± 61	45 ± 7	**Ca**	16,100 ± 5040	26,100 ± 8030	270 ± 50	33 ± 5
**Na**	790 ± 230	33,100 ± 10,300	6.6 ± 1.3	4.2 ± 0.6	**Na**	143 ± 41	17,200 ± 5040	3.0 ± 0.6	6.6 ± 1.0
**Mg**	190 ± 62	6210 ± 1920	130 ± 25	65 ± 10	**Mg**	161 ± 52	5040 ± 1510	100 ± 20	58 ± 9
**K**	733 ± 221	9310 ± 2820	2460 ± 492	940 ± 140	**K**	780 ± 230	5830 ± 1700	2410 ± 480	1561 ± 234
**Al**	31 ± 11	2910 ± 9210	4.0 ± 0.8	0.214 ± 0.032	**Al**	42 ± 10	19,100 ± 6030	1.6 ± 0.3	0.350 ± 0.053
**Fe**	4.0 ± 1.2	24,100 ± 7240	5.0 ± 1.0	3.480 ± 0.520	**Fe**	6.0 ± 2.0	22,300 ± 7020	5.1 ± 1.0	1.510 ± 0.227
**Ba**	16 ± 5	80 ± 24	1.5 ± 0.3	0.026 ± 0.004	**Ba**	33 ± 10	91 ± 27	1.2 ± 0.2	0.044 ± 0.007
**Mn**	24 ± 7	450 ± 135	2.0 ± 0.4	1.250 ± 0.190	**Mn**	60 ± 18	653 ± 196	1.7 ± 0.3	1.500 ± 0.225
**Sr**	23 ± 6	120 ± 36	2.5 ± 0.5	0.760 ± 0.115	**Sr**	245 ± 70	93 ± 28	1.3 ± 0.3	0.525 ± 0.079
**Cd**	0.06 ± 0.02	0.26 ± 0.08	<MLOQ	<MLOQ	**Cd**	0.09 ± 0.03	0.45 ± 0.14	<MLOQ	<MLOQ
**Co**	0.05 ± 0.02	5.8 ± 1.7	0.01 ± 0.002	<MLOQ	**Co**	0.06 ± 0.02	8.2 ± 2.5	<MLOQ	<MLOQ
**Cr**	0.17 ± 0.05	147 ± 44	<MLOQ ^3^	<MLOQ	**Cr**	0.21 ± 0.06	99 ± 29	0.016 ± 0.003	<MLOQ
**Li**	0.16 ± 0.05	22 ± 6	0.010 ± 0.002	0.010 ± 0.002	**Li**	0.20 ± 0.06	13 ± 4	0.009 ± 0.002	0.011 ± 0.002
**Rb**	1.9 ± 0.6	71 ± 21	3.6 ± 0.7	1.950 ± 0.290	**Rb**	1.3 ± 0.4	37 ± 11	3.5 ± 0.7	1.250 ± 0.188
**Ti**	0.19 ± 0.06	296 ± 89	1.3 ± 0.3	0.042 ± 0.006	**Ti**	0.16 ± 0.05	137 ± 41	0.80 ± 0.15	0.032 ± 0.005
**V**	0.13 ± 0.04	85 ± 25	0.040 ± 0.007	<MLOQ	**V**	0.18 ± 0.05	52 ± 16	0.024 ± 0.005	<MLOQ
**Ni**	0.86 ± 0.26	22 ± 7	0.071 ± 0.014	0.154 ± 0.023	**Ni**	1.3 ± 0.4	26 ± 8	0.024 ± 0.005	0.066 ± 0.010
**Cu**	2.2 ± 0.7	47 ± 14	1.2 ± 0.2	0.038 ± 0.006	**Cu**	2.6 ± 0.8	46 ± 14	0.87 ± 0.17	0.116 ± 0.017
**Pb**	0.70 ± 0.21	3.6 ± 1.1	<MLOQ	0.079 ± 0.012	**Pb**	0.50 ± 0.15	4.5 ± 1.4	0.03 ± 0.01	0.080 ± 0.012
**Zn**	0.57 ± 0.17	42 ± 13	1.1 ± 0.2	0.275 ± 0.041	**Zn**	0.59 ± 0.18	46 ± 14	1.2 ± 0.2	0.255 ± 0.038

^1^ MF—mobile-form element. ^2^ TC—total content element. ^3^ *MLOQ—method limit of quantification.*

**Table 3:** Data on the elemental composition of soils, grapes, and wine, classified by their qualitative and quantitative phase composition as group 3.

Element	Haykadzor				Element	Gostagaevskaya			
	MF ^1^, mg/kg	TC ^2^, mg/kg	Grape, mg/kg	Wine, mg/L		MF ^1^, mg/kg	TC ^2^, mg/kg	Grape, mg/kg	Wine, mg/L
**Ca**	62,100 ± 18,100	103,200 ± 30,300	200 ± 40	53 ± 8	**Ca**	12,100 ± 4020	40,300 ± 12,200	250 ± 50	58 ± 9
**Na**	42 ± 11	7910 ± 2410	3.0 ± 0.6	25 ± 4	**Na**	112 ± 34	9530 ± 2840	6.2 ± 1.2	8.0 ± 1.3
**Mg**	274 ± 83	3220 ± 930	110 ± 20	92 ± 14	**Mg**	641 ± 193	8020 ± 2410	110 ± 2	60 ± 9
**K**	371 ± 112	6740 ± 2130	1560 ± 310	1131 ± 170	**K**	322 ± 104	5810 ± 1700	2150 ± 430	910 ± 140
**Al**	41 ± 11	24,100 ± 7230	2.4 ± 0.5	0.310 ± 0.047	**Al**	24 ± 7	27,300 ± 8120	3.0 ± 0.6	0.445 ± 0.067
**Fe**	4.0 ± 1.1	21,300 ± 6120	5.7 ± 1.1	1.600 ± 0.240	**Fe**	10 ± 3	27,300 ± 8020	5.2 ± 1.0	4.520 ± 0.678
**Ba**	50 ± 15	114 ± 34	1.4 ± 0.3	0.078 ± 0.012	**Ba**	45 ± 14	112 ± 33	1.5 ± 0.3	0.081 ± 0.012
**Mn**	85 ± 26	800 ± 240	1.9 ± 0.4	0.790 ± 0.119	**Mn**	65 ± 20	634 ± 190	0.90 ± 0.17	0.570 ± 0.086
**Sr**	47 ± 14	72 ± 22	0.62 ± 0.12	0.655 ± 0.098	**Sr**	96 ± 29	190 ± 57	3.0 ± 0.6	0.785 ± 0.118
**Cd**	0.07 ± 0.02	0.36 ± 0.11	<MLOQ	<MLOQ	**Cd**	0.12 ± 0.04	0.36 ± 0.11	<MLOQ	<MLOQ
**Co**	0.12 ± 0.04	8.4 ± 2.5	<MLOQ	<MLOQ	**Co**	0.07 ± 0.02	11 ± 3	<MLOQ	<MLOQ
**Cr**	0.27 ± 0.08	55 ± 17	<MLOQ	<MLOQ	**Cr**	0.21 ± 0.06	55 ± 16	<MLOQ	<MLOQ
**Li**	0.40 ± 0.12	16 ± 5	0.007 ± 0.002	0.0068 ± 0.0010	**Li**	0.4 ± 0.12	18 ± 5	0.014 ± 0.003	0.013 ± 0.002
**Rb**	<MLOQ	55 ± 16	2.1 ± 0.4	1.550 ± 0.233	**Rb**	1.8 ± 0.5	49 ± 14	2.0 ± 0.4	1.395 ± 0.210
**Ti**	<MLOQ	350 ± 105	1.0 ± 0.2	0.039 ± 0.0056	**Ti**	0.13 ± 0.04	110 ± 34	1.0 ± 0.2	0.041 ± 0.006
**V**	0.10 ± 0.03	48 ± 14	0.034 ± 0.007	0.00010 ± 0.00002	**V**	0.22 ± 0.07	66 ± 20	0.030 ± 0.006	0.00095 ± 0.00014
**Ni**	1.4 ± 0.4	27 ± 8	0.013 ± 0.003	0.075 ± 0.011	**Ni**	1.9 ± 0.6	42 ± 12	0.023 ± 0.005	0.196 ± 0.029
**Cu**	2.8 ± 0.8	48 ± 14	0.74 ± 0.15	0.120 ± 0.018	**Cu**	0.80 ± 0.24	50 ± 15	0.82 ± 0.16	<MLOQ
**Pb**	1.3 ± 0.4	8.6 ± 2.6	0.03 ± 0.01	0.0046 ± 0.0007	**Pb**	0.98 ± 0.29	6.2 ± 1.9	0.06 ± 0.01	0.056 ± 0.008
**Zn**	0.58 ± 0.17	53 ± 15	0.80 ± 0.16	0.076 ± 0.011	**Zn**	1.0 ± 0.3	64 ± 19	1.1 ± 0.2	0.101 ± 0.015

^1^ MF—mobile-form element. ^2^ TC—total content element. ^3^ *MLOQ—method limit of quantification.*

**Table 4:** Migration of elements from soil to grapes.

Element	Biologic Absorption Coefficient					
	Yurovka	Vinogradny	Raevskaya	Anapa	Haykadzor	Gostagaevskaya
Ca	0.073	0.069	0.057	0.016	0.003	0.020
Na	0.031	0.266	0.008	0.021	0.072	0.054
Mg	0.226	0.290	0.639	0.605	0.390	0.177
K	16.14	28.81	3.26	3.10	3.68	6.89
Al	0.060	0.045	0.124	0.046	0.073	0.125
Fe	0.628	1.04	1.43	0.859	1.41	0.533
Ba	0.039	0.032	0.095	0.037	0.030	0.034
Mn	0.048	0.057	0.083	0.029	0.023	0.013
Sr	0.130	0.168	0.116	0.055	0.013	0.032
Cd	-	0.025	-	-	-	-
Co	0.035	0.093	0.161	0.083	0.029	0.041
Cr	0.060	0.153	0.072	0.077	0.026	0.044
Li	0.040	0.041	0.061	0.045	0.019	0.035
Rb	3.85	0.812	1.81	2.63	2.38	1.13
Ti	2.50	3.50	6.61	4.58	-	7.86
V	0.734	0.374	0.321	0.146	0.355	0.137
Ni	0.023	0.022	0.086	0.017	0.009	0.012
Cu	2.790	2.272	0.518	0.329	0.305	0.993
Pb	0.007	0.005	0.003	0.005	0.002	0.006
Zn	3.94	5.02	2.01	1.95	1.47	1.07

**Table 5:** Correlation coefficients (R^2^) between the concentrations of elements in the soil–berry chain depending on the depth of soil sampling.

Vineyard	Mobile-Form Element							
	0–20 cm	20–40 cm	40–60 cm	60–80 cm	80–100 cm	100–120 cm	120–140 cm	140–160 cm
Yurovka	0.09092	0.09286	0.08572	0.08476	0.08657	0.08425	0.08459	0.08129
Gostagaevskaya	0.08026	0.07789	0.07832	0.07036	0.06741	0.06600	0.06103	0.06044
Anapa	0.11353	0.10647	0.09680	0.06388	0.05525	0.05291	0.05276	0.05272
Raevskaya	0.21476	0.22038	0.19200	0.12604	0.12152	0.07454	0.09389	0.08288
Haykadzor	0.08795	0.08883	0.08854	0.08707	0.08574	0.08578	0.08578	0.08413
Vinogradny	0.06069	0.05063	0.03776	0.04089	0.03510	0.02883	0.01994	0.01312
**Vineyard**	**Total Content Element**							
	**0–20 cm**	**20–40 cm**	**40–60 cm**	**60–80 cm**	**80–100 cm**	**100–120 cm**	**120–140 cm**	**140–160 cm**
Yurovka	−0.04702	−0.01178	0.00226	0.00524	0.05104	0.01698	0.05056	0.00527
Gostagaevskaya	0.07781	0.11265	0.07356	0.10395	0.09876	0.07908	0.06077	0.08421
Anapa	0.12944	0.12031	0.08406	0.08234	0.04431	0.06064	0.06226	0.04291
Raevskaya	0.08311	0.09205	0.08926	0.06110	0.06643	0.09196	0.05079	0.08665
Haykadzor	0.12699	0.13044	0.11108	0.12745	0.10515	0.11488	0.11254	0.10067
Vinogradny	0.06250	0.13935	0.12474	0.10305	0.02809	0.09284	0.10190	0.06780

**Table 6:** Results of discriminant analysis of wine samples.

*N* = 90	Discriminant Function Analysis Summary No. of Vars in Model: 19; Grouping: Region (6 Grps) Wilks’ Lambda: 0.00000; Approx. F (45.343) = 446.55 *p* < 0.000					
	Wilks’ Lambda	Partial Lambda	F-Remove (2.78)	*p*-Value	Tolerance	1-Tolerance (R-Sqr.)
**Rb**	1.837 × 10^−7^	0.062	228.136	0.000 × 10^−1^	0.897	0.103
**Al**	4.613 × 10^−8^	0.249	45.900	1.339 × 10^−21^	0.821	0.179
**K**	4.268 × 10^−8^	0.269	41.339	2.455 × 10^−20^	0.822	0.178
**Sr**	3.245 × 10^−8^	0.354	27.789	6.828 × 10^−16^	0.667	0.333
**Co**	3.197 × 10^−8^	0.359	27.154	1.188 × 10^−15^	0.756	0.244
**Na**	2.682 × 10^−8^	0.428	20.324	8.054 × 10^−13^	0.738	0.262
**Pb**	2.018 × 10^−8^	0.569	11.527	2.675 × 10^−8^	0.852	0.148
**Ca**	2.004 × 10^−8^	0.573	11.341	3.441 × 10^−8^	0.754	0.246
**Ni**	1.969 × 10^−8^	0.583	10.877	6.503 × 10^−8^	0.894	0.106

**Table 7:** Physico-chemical properties of samples of Cabernet Sauvignon wines.

The Indicator Being Determined	Territory					
	Raevskaya	Anapa	Haykadzor	Vinogradny	Gostagaevskaya	Yurovka
Alcohol content, (%, *v*/*v*)	10.9	12.1	11.7	9.1	9.7	10.9
Titratable acidity, (g/L)	5.3	4.8	6.3	10.8	9.6	7.2
Volatile acidity, (g/L)	0.53	0.60	0.59	0.53	0.52	0.56
Total sugar, (g/L)	1.0	2.4	2.3	1.5	1.6	0.54
pH	3.6	3.8	3.5	3.3	3.2	3.1
Total phenolic, (mg/mL)	1793	2493	2710	1388	1458	1676
Total SO_2_, (mg/L)	95	92	86	88	78	66

**Table 8:** Instrument operating parameters and element limits of quantification.

ICP-OES (iCAP 7400)			
Plasma gas flow rate, L/min		12.0	
Nebulizer gas flow rate, L/min		0.5	
Auxiliary gas flow rate, L/min		0.5	
Applied power, W		1200	
**Spectral lines of elements (LOQ ^1^, µg/L)**			
Al 396.152 (I), (1.13)	Cu 324.754 (I), (1.41)		Ni 231.604 (II), (0.76)
As 189.042 (I), (2.7)	Fe 259.940 (II), (1.34)		Pb 220.353 (II), (1.88)
Ba 455.403 (II), (0.41)	K 766.490 (I), (5.2)		Rb 780.023 (I), (0.72)
Ca 422.673 (I), (14)	Li 670.784 (I), (0.26)		Sr 421.552 (II), (0.57)
Cd 226.502 (II), (0.24)	Mg 280.270 (II), (6.0)		Ti 334.941 (II), (1.6)
Co 238.892 (II), (0.79)	Mn 257.610 (II), (0.35)		V 292.402 (II), (0.74)
Cr 267.716 (II), (0.89)	Na 588.995 (I), (2.4)		Zn 213.856 (I), (0.85)

^1^ LOQ = 10σ_0_, where σ0 is the standard deviation of blank results.

**Table 9:** Results of analysis of standard soil samples.

Elements	Element Content, mg/kg			
	Mobile Form (SADPP 10)		Total Content (SDPS-2)	
	Certified Value	Found	Certified Value	Found
Cu	0.18 ± 0.01	0.21 ± 0.06	100 ± 10	101 ± 30
Zn	1.38 ± 0.06	1.40 ± 0.42	140 ± 20	136 ± 41
Cd	0.058 ± 0.002	0.055 ± 0.017	1.3 ± 0.3	1.3 ± 0.4
Pb	0.66 ± 0.02	0.70 ± 0.21	87 ± 5	84 ± 25
Ni	0.61 ± 0.02	0.60 ± 0.18	87 ± 9	88 ± 26
Co	0.13 ± 0.01	0.12 ± 0.04	45 ± 3	44 ± 13
Mn	31.3 ± 1.6	30.0 ± 9.0	-	-

## Data Availability

All data are included in the article.
